# The original sins of clinical trials with intravenous immunoglobulins in sepsis

**DOI:** 10.1186/s13054-015-0793-0

**Published:** 2015-02-26

**Authors:** Raquel Almansa, Eduardo Tamayo, David Andaluz-Ojeda, Leonor Nogales, Jesús Blanco, Jose Maria Eiros, Jose Ignacio Gomez-Herreras, Jesus F Bermejo-Martin

**Affiliations:** Group of Biomedical Research in Critical Care Medicine (BioCritic), Hospital Clínico Universitario de Valladolid, Avda. Ramón y Cajal, 3. 47005, Valladolid, Spain; Servicio de Medicina Intensiva, Hospital Universitario Rio Hortega, Calle Dulzaina, 2, 47012, Valladolid, Spain; Centro de Investigación en Red de Enfermedades Respiratorias (CIBERES), Instituto de Salud Carlos III, Calle Sinesio Delgado, 4, 28029, Madrid, Spain

## Abstract

Intravenous immunoglobulins (IVIGs) have not yet demonstrated robust evidence in the benefit for treatment of sepsis. In spite of multiple clinical trials performed with IVIG in sepsis, it remains an experimental therapy for this severe condition. Nonetheless, these trials do not address a number of potential confounding factors, concerning both the patient and the IVIG preparations, which could greatly affect the final result. To name a few, endogenous levels of immunoglobulin isotypes and subclasses are not assessed prior to treatment. The presence/absence of patient antibodies against the microorganism(s) causing sepsis is not evaluated. The accuracy of antibiotic prescription is not included as an adjusting variable. The degree of patient immunosuppression (previous or induced by sepsis) is not documented. In turn, the concentration and antimicrobial specificities of the antibodies contained in the batches of IVIG are not assessed. Neither the pharmacokinetics of IVIG nor its potential immunomodulatory effects are evaluated. In addition, the concept of ‘window of opportunity’ for IVIG administration following diagnosis of sepsis is not considered. In conclusion, addressing these factors could help to individualise treatment with IVIG for sepsis, which could enhance the opportunities of this drug to show benefits in terms of survival in this severe condition.

## Introduction

**Figure 1:**
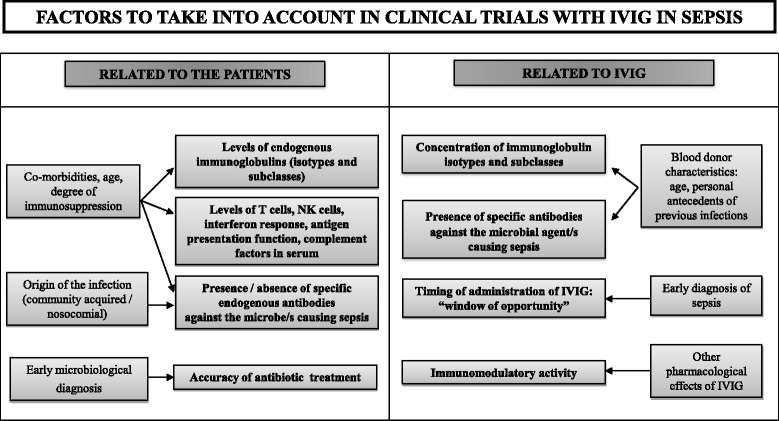
**Potential confounding factors not appropriately considered in the design or analysis of the clinical trials evaluating IVIG for the treatment of sepsis.** Main factors are in bold. Other factors related to them are in standard type. IVIG, intravenous immunoglobulin; NK, natural killer.

Mortality associated with severe sepsis and septic shock ranges from 20% to 30% [1]. Many different approaches tested for treating this disease failed to improve survival [1]. The presence of low levels of immunoglobulins (Igs) in serum is a frequent finding in severe sepsis and septic shock, ranging from 25% to 61% of the patients in the case of IgG and 19% to 33% for IgM [2-4]. Nonetheless, results from clinical trials evaluating exogenous Igs for treatment of this disease are controversial [5,6]. A meta-analysis published in 2013 concluded that there was a protective effect of polyclonal intravenous immunoglobulins (IVIGs) against mortality among adults with sepsis, which was not seen in trials with low risk of bias [5]. In contrast, Kreymann and colleagues [7] reported that the mortality-reducing effect of IVIG was also seen in trials with the highest methodological quality. In our view, clinical trials assessing IVIG for the treatment of sepsis do not appropriately address a number of important factors that could greatly affect the final result (Figure 1).

## Factors related to the patient

### The influence of endogenous levels of immunoglobulins

The influence of endogenous levels of Igs on prognosis remains unclear. Whereas the SBITS (Score-Based Immunoglobulin Therapy of Sepsis) study [8] did not show any negative influence of a low IgG level on 28-day mortality [2], Taccone and colleagues [9] found that patients with community-acquired septic shock and hypo-IgG had higher mortality. Similarly, Průcha and colleagues [10] reported the presence of IgG hypogammaglobulinemia in patients with severe sepsis as an independent factor of mortality. In turn, we have recently demonstrated the existence of a statistical association between low endogenous levels of Igs and poor outcome in severe sepsis and septic shock [4]. Differences among studies could be explained in part by the different composition of the patient cohorts. In the SBITS study, percentile 25 for IgG distribution was 610 mg/dL, whereas in our study it was 374 mg/dL, probably because of a higher frequency of patients with prior immunosuppressive conditions in our cohort (Werdan K, personal communication). Although it remains to be elucidated whether low levels of endogenous Igs are a marker of disease severity rather than a cause of mortality, our opinion is that it is a potential confounding factor that has to be taken into account in the IVIG trials. In addition, the distinct Ig subclasses and isotypes (IgG1, IgM, and IgA) have a different influence on prognosis in sepsis [4]. Combined deficits of these isotypes are associated with higher risk of mortality [4]. Assessing levels of basal Ig isotypes could help us to understand the potential effect of IVIG preparations containing only IgG and of those enriched in IgM or IgA or both. It could be also important to evaluate the presence/absence of pre-existing specific antibodies against the microbe causing the septic insult [11].

### Accuracy of antibiotic prescription

Early administration of an appropriate antibiotic treatment, along with life support measures, is the only treatment that has been demonstrated to improve survival in sepsis [1]. Therefore, the accuracy of antibiotic treatment could greatly influence the results of IVIG. In this sense, the application of new proteomics and genomics methods is translating into an earlier and more exact microbiological diagnosis, which in turn will help to optimise antibiotic prescription in sepsis [12].

### Evaluation of the basal degree of immunosuppression

Sepsis is often associated with the presence of immunosuppression. This condition could greatly affect levels of endogenous Igs but also levels or function of other elements of the immune system which are very important for achieving infection control (antigen presentation, interferon response, T cells, natural killer cells, neutrophils, and complement factors) [13,14]. Quantitative and qualitative evaluation of these elements could help to obtain a good picture of the basal immunological status of the patient receiving IVIG.

## Factors related to intravenous immunoglobulin

### Intravenous immunoglobulin composition

IVIG content in Ig isotypes could influence trials results. This way, IgM-enriched IVIG could render more benefits in terms of survival than those preparations containing just IgG [5,7]. Another major issue is the absence of evidence characterising the primary therapeutic principles in IVIG (Fab elements ?, Fc fragments ?) which could have potentially beneficial effects in sepsis (toxin scavenging, antibacterial effect, and immunomodulation) [6]. Moreover, the concentration and antimicrobial specificities of the antibodies contained in the IVIG preparations are not taken sufficiently into account. These are highly dependent on the personal antecedents of natural infections and vaccination of the blood donors for IVIG, who are healthy individuals. In consequence, IVIG could be deficient in antibodies against microbes causing hospital-acquired infections. Developing new IVIG preparations obtained from pools of sera of survivors to sepsis of either community or nosocomial origin could solve this problem. In addition, taking into account the age of the blood donors could be important for understanding the biological properties of IVIG since the natural IgG Ig repertoire is highly dependent on age.

### Dosage, timing, and pharmacokinetics of intravenous immunoglobulin

There is probably a ‘window of opportunity’ for IVIG in the first days that follow clinical presentation of sepsis [15]. If this window is missed, probabilities of success could be greatly diminished. In addition, monitoring Ig levels along the course of the treatment would help us to understand the pharmacokinetics of IVIG in patients with sepsis which is relevant for further dosage calculation. In turn, it is unknown whether the main goal of IVIG in sepsis has to be to refill low levels of endogenous Igs or alternatively whether IVIG could exert a beneficial effect independently of these levels. Some of the factors listed above are difficult to assess at the moment a patient is included into a clinical trial but could be assessed afterwards, in the phase of data analysis.

## Conclusions

Addressing the factors exposed here could help us to individualise treatment with IVIG for sepsis and in turn to enhance the opportunities of this treatment to show benefits in terms of survival in this severe condition.

## Abbreviations

Ig: Immunoglobulin
IVIG: Intravenous immunoglobulin
SBITS: Score-Based Immunoglobulin Therapy of Sepsis

## References

[1] Angus DC, van der Poll T (2013). Severe sepsis and septic shock. N Engl J Med..

[2] Päsler M, Dietz S, Werdan K, Vincent PJ-L (2012). Hypogammaglobulinemia in Sepsis. Annual Update in Intensive Care and Emergency Medicine 2012.

[3] Venet F, Gebeile R, Bancel J, Guignant C, Poitevin-Later F, Malcus C (2011). Assessment of plasmatic immunoglobulin G, A and M levels in septic shock patients. Int Immunopharmacol..

[4] Bermejo-Martín JF, Rodriguez-Fernandez A, Herrán-Monge R, Andaluz-Ojeda D, Muriel-Bombín A, Merino P (2014). Immunoglobulins IgG1, IgM and IgA: a synergistic team influencing survival in sepsis. J Intern Med..

[5] Alejandria MM, Lansang MAD, Dans LF, Mantaring JB (2013). Intravenous immunoglobulin for treating sepsis, severe sepsis and septic shock. Cochrane Database Syst Rev..

[6] Shankar-Hari M, Spencer J, Sewell WA, Rowan KM, Singer M (2012). Bench-to-bedside review: immunoglobulin therapy for sepsis - biological plausibility from a critical care perspective. Crit Care..

[7] Kreymann KG, de Heer G, Nierhaus A, Kluge S (2007). Use of polyclonal immunoglobulins as adjunctive therapy for sepsis or septic shock. Crit Care Med..

[8] Werdan K, Pilz G, Bujdoso O, Fraunberger P, Neeser G, Schmieder RE (2007). Score-based immunoglobulin G therapy of patients with sepsis: the SBITS study. Crit Care Med..

[9] Taccone FS, Stordeur P, De Backer D, Creteur J, Vincent J-L (2009). Gamma-globulin levels in patients with community-acquired septic shock. Shock Augusta Ga..

[10] Průcha M, Zazula R, Herold I, Dostál M, Hyánek T, Bellingan G (2013). Presence of hypogammaglobulinemia - a risk factor of mortality in patients with severe sepsis, septic shock, and SIRS. Prague Med Rep..

[11] Adhikari RP, Ajao AO, Aman MJ, Karauzum H, Sarwar J, Lydecker AD (2012). Lower antibody levels to Staphylococcus aureus exotoxins are associated with sepsis in hospitalized adults with invasive S. aureus infections. J Infect Dis.

[12] Liesenfeld O, Lehman L, Hunfeld K-P, Kost G (2014). Molecular diagnosis of sepsis: new aspects and recent developments. Eur J Microbiol Immunol..

[13] Andaluz-Ojeda D, Iglesias V, Bobillo F, Almansa R, Rico L, Gandía F (2011). Early natural killer cell counts in blood predict mortality in severe sepsis. Crit Care..

[14] Bermejo-Martín JF, Tamayo E, Ruiz G, Andaluz-Ojeda D, Herrán-Monge R, Muriel-Bombín A (2014). Circulating neutrophil counts and mortality in septic shock. Crit Care..

[15] Berlot G, Vassallo MC, Busetto N, Bianchi M, Zornada F, Rosato I (2012). Relationship between the timing of administration of IgM and IgA enriched immunoglobulins in patients with severe sepsis and septic shock and the outcome: a retrospective analysis. J Crit Care..

